# Deep coupled registration and segmentation of multimodal whole-brain images

**DOI:** 10.1093/bioinformatics/btae606

**Published:** 2024-10-14

**Authors:** Tingting Han, Jun Wu, Pengpeng Sheng, Yuanyuan Li, ZaiYang Tao, Lei Qu

**Affiliations:** Ministry of Education Key Laboratory of Intelligent Computation and Signal Processing, Information Materials and Intelligent Sensing Laboratory of Anhui Province, School of Electronics and Information Engineering, Anhui University, Hefei, Anhui, 230601, China; Ministry of Education Key Laboratory of Intelligent Computation and Signal Processing, Information Materials and Intelligent Sensing Laboratory of Anhui Province, School of Electronics and Information Engineering, Anhui University, Hefei, Anhui, 230601, China; Ministry of Education Key Laboratory of Intelligent Computation and Signal Processing, Information Materials and Intelligent Sensing Laboratory of Anhui Province, School of Electronics and Information Engineering, Anhui University, Hefei, Anhui, 230601, China; Ministry of Education Key Laboratory of Intelligent Computation and Signal Processing, Information Materials and Intelligent Sensing Laboratory of Anhui Province, School of Electronics and Information Engineering, Anhui University, Hefei, Anhui, 230601, China; Ministry of Education Key Laboratory of Intelligent Computation and Signal Processing, Information Materials and Intelligent Sensing Laboratory of Anhui Province, School of Electronics and Information Engineering, Anhui University, Hefei, Anhui, 230601, China; Ministry of Education Key Laboratory of Intelligent Computation and Signal Processing, Information Materials and Intelligent Sensing Laboratory of Anhui Province, School of Electronics and Information Engineering, Anhui University, Hefei, Anhui, 230601, China; SEU-ALLEN Joint Center, Institute for Brain and Intelligence, Southeast University, Nanjing, Jiangsu, 210096, China; Institute of Artiffcial Intelligence, Hefei Comprehensive National Science Center, Hefei, 231299, China; Hefei National Laboratory, University of Science and Technology of China, Hefei, 230094, China

## Abstract

**Motivation:**

Recent brain mapping efforts are producing large-scale whole-brain images using different imaging modalities. Accurate alignment and delineation of anatomical structures in these images are essential for numerous studies. These requirements are typically modeled as two distinct tasks: registration and segmentation. However, prevailing methods, fail to fully explore and utilize the inherent correlation and complementarity between the two tasks. Furthermore, variations in brain anatomy, brightness, and texture pose another formidable challenge in designing multi-modal similarity metrics. A high-throughput approach capable of overcoming the bottleneck of multi-modal similarity metric design, while effective leveraging the highly correlated and complementary nature of two tasks is highly desirable.

**Results:**

We introduce a deep learning framework for joint registration and segmentation of multi-modal brain images. Under this framework, registration and segmentation tasks are deeply coupled and collaborated at two hierarchical layers. In the inner layer, we establish a strong feature-level coupling between the two tasks by learning a unified common latent feature representation. In the outer layer, we introduce a mutually supervised dual-branch network to decouple latent features and facilitate task-level collaboration between registration and segmentation. Since the latent features we designed are also modality-independent, the bottleneck of designing multi-modal similarity metric is essentially addressed. Another merit offered by this framework is the interpretability of latent features, which allows intuitive manipulation of feature learning, thereby further enhancing network training efficiency and the performance of both tasks. Extensive experiments conducted on both multi-modal and mono-modal datasets of mouse and human brains demonstrate the superiority of our method.

**Availability and implementation:**

The code is available at https://github.com/tingtingup/DCRS.

## 1 Introduction

A major challenge in modern neuroscience is comprehending the structure and function of mammalian brain. Recent advances in high-resolution light microscopy, tissue clearing, and sparse labeling techniques have rendered the mapping of mammalian whole-brain at single-cell resolution a feasible prospect. Several large international efforts have been initiated (Hintiryan *et al.* 2016, Ecker *et al.* 2017), resulting in the accumulation of large-scale whole-brain images at unprecedented speeds. These images are often acquired using different imaging techniques, including serial two photon tomography (STPT) (Economo *et al.* 2016), fluorescence micro-optical sectioning tomography (fMOST) (Gong *et al.* 2016), light-sheet fluorescence microscopy (LSFM) (Dodt *et al.* 2007) or volumetric imaging with synchronous on-the-fly-scan and readout (VISoR) (Xu *et al.* 2021). Two enabling computational techniques to interpret these data are registration and segmentation. Image registration involves mapping the coordinates of the moving image onto those of a fixed image through elastic space transformations, thereby facilitating the comparison, analysis, and visualization of brain data from different individuals, developmental stages, scales, and imaging modalities in a unified coordinate space. Brain segmentation, on the other hand, distinguishes and delineates boundaries of different brain regions based on anatomical priors, imaging intensity, as well as differences in cellular morphology and density. The accurate registration and segmentation of brain imaging data are crucial for brain atlas building, neuron type identification, and analysis of neural connections, neural projections, and gene expression.

Due to the substantial disparities in input and output characteristics between registration and segmentation, such as differences in input image count, output dimensions, and types, they are typically regarded as separated topics in computer vision, medical imaging, and biological image processing domains (Wang *et al.* 2022). However, these two tasks could be highly correlated and complementary. In the context of atlas-based registration, brain segmentation can be achieved by inversely mapping the annotation template of an anatomical atlas (e.g., the mouse CCFv3; Wang *et al.* 2020) using the displacement field obtained from registration. Conversely, segmentation-based registration involves initially delineating the boundaries of the brain regions either automatically or manually, followed by aligning these boundaries to perform image registration. Moreover, segmentation simplifies the image representation, encouraging registration to focus on aligning the boundaries of regions of interest. While atlas-based registration, through deforming the anatomical template, provides shape and relative position constraints for improved segmentation. Effective coordination of these tasks in a coupled and collaborative manner could potentially enhance the performance of both tasks simultaneously.

Several deep learning-based methods have emerged to explore the correlation between registration and segmentation tasks. One category of methods utilizes the automatic segmentation results to provide weak supervision for registration (Xu and Niethammer 2019, He *et al.* 2020, Qiu and Ren 2021). While these methods leverage supervision data from both tasks in a complementary manner, they often overlook the intrinsic correlation between them. Another category involves concatenating the fixed and moving images as input to the network and attempting to mine inter-task correlations using a shared coding layer (Estienne *et al.* 2019). However, since registration and segmentation involve different numbers of input images, simply concatenating the images will lead to significant parameter redundancy and increase the training difficulty. In addition, neither of the aforementioned methods adequately consider the challenges of multi-modal scenarios. The significant differences in brightness, texture, and structure in whole-brain images of different modalities render similarity measures (e.g., mean squared error. MSE) (Bauer and Kohavi 1999) used in the above methods no longer applicable.

Current deep learning-based multi-modal image registration methods can be broadly classified into two categories. One category utilizes image similarity metrics based on information theory, such as mutual information (MI), normalized mutual information (NMI) to calculate the misalignment between images. However, these metrics often suffer from local anatomy and intensity variations (Woo *et al.* 2014), which are common in biological *ex vivo* imaging. Another category of methods attempts to convert multi-modal registration tasks into mono-modal ones by using image-to-image translation. For instance, Arar *et al.* (2020) first translate one image modality to another using generative adversarial networks (GANs), and then perform image registration within the same modality. However, this approach is susceptible to registration failure as the image structure may not be preserved during image translation without additional constraints. Qin *et al.* (2019) decompose images of different modalities into a common latent shape space and separate latent appearance spaces using an unsupervised translation approach, and then conducts registration in latent shape space. Nonetheless, the disentangled network lacks interpretability, which is crucial for multi-modal registration tasks due to the intricate relationship between multi-modal images (Deng *et al.* 2023).

In this article, we propose a novel deep learning-based framework, called Deep Coupled Registration and Segmentation (DCRS) of multi-modal whole-brain images. We realize the feature-level coupling between the two tasks by learning a unified modality-independent latent feature representation. This allows seamless integration of information across tasks and modalities while inherently circumventing the challenge of designing robust multi-modal similarity metrics. We further exploit the complementarity of the two tasks and achieve their task-level collaboration by designing a mutual supervised dual-branch network. In addition, we introduce an Exponential Signed Distance Representation (ESDR) and a two-stage training scheme for registration and segmentation to cultivate and utilize the interpretability of latent features, thereby further improving the performance and learning efficiency of our model. Experimental results on mouse brains demonstrate the superiority of our method over state-of-the-art multi-modal registration techniques, while yielding satisfactory segmentation results. We also verify the good generalization of our method on human brain datasets.

## 2 Materials and methods

There are two aspects in the DCRS framework: (i) Given the disparities in input and output between registration and segmentation, how to construct a deep network architecture that can achieve strong inner-layer coupling and deep outer-layer collaboration between the two tasks (Section 2.1). (ii) How to effectively guide and drive the training of the network so that it can efficiently learn modality-independent common latent feature representations suitable for both registration and segmentation tasks (Section 2.2).

### 2.1 Deep coupled registration and segmentation

**Figure 1.**
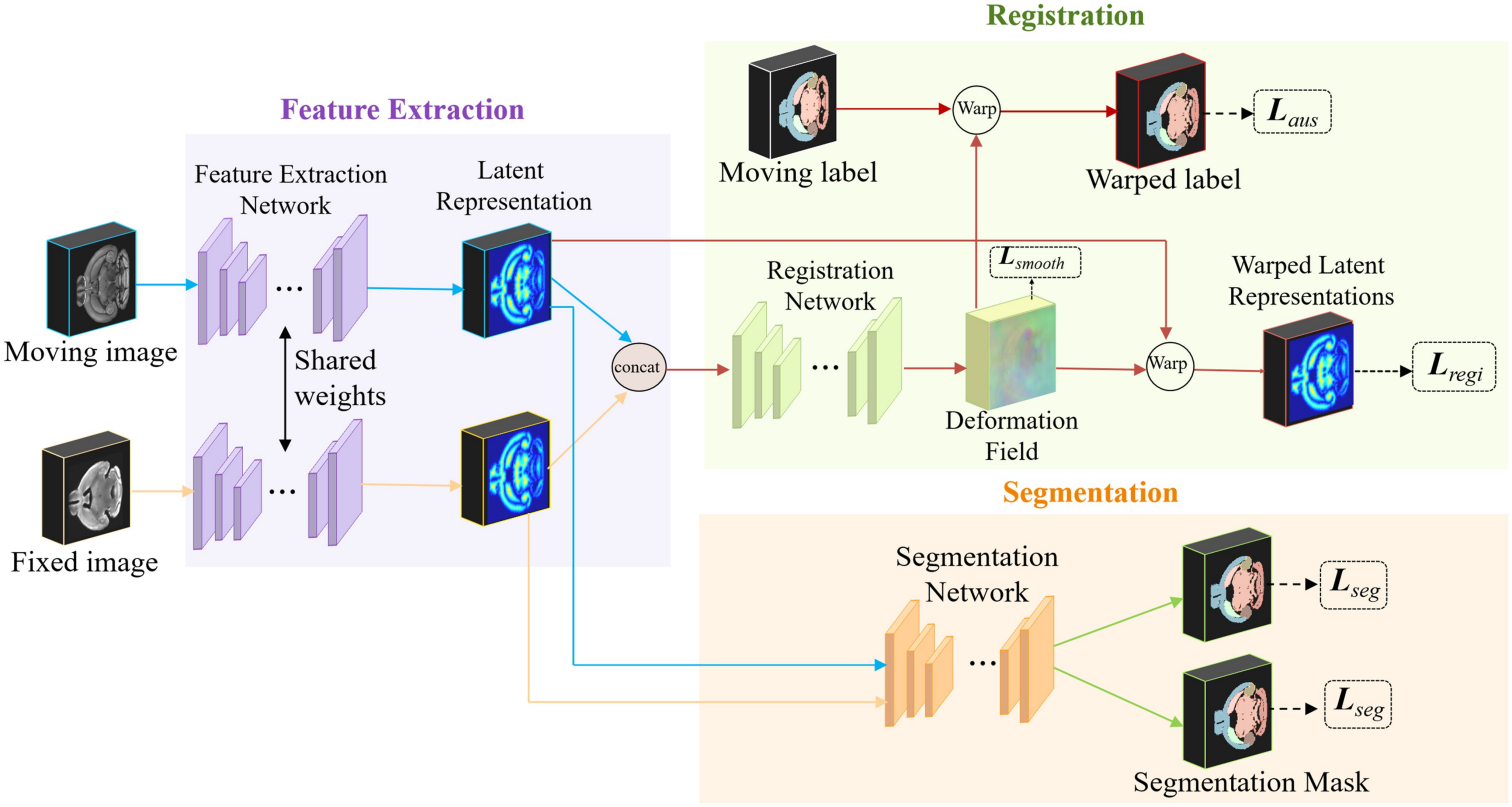
An overview of DCRS framework. The DCRS framework contains three modules: feature extraction network G, registration network R, and segmentation network S. The three modules of DCRS form two branches, where G-S constitutes the segmentation branch and G–R is the registration branch. An ESDR-guided two-stage training scheme is introduced to encourage the task commonality, modality independence, and interpretability of learned latent features.

As illustrated in Fig. 1, the DCRS framework comprises three modules: the feature extraction network G, the segmentation network S, and the registration network R. The fixed and moving images are 3D single-channel grayscale volumes, and can be of the same or different modalities. Network G is designed to extract modality-independent common latent features from moving images and fixed images. The extracted image features of different images are then individually or pairwise fed into the segmentation and registration networks, facilitating the feature sharing and deep coupling between the two tasks at the feature level. The three modules of DCRS form two branches, where G-S constitutes the segmentation branch and G-R is the registration branch. The G-R branch takes paired images as input and generates displacement field and warped images, while the G-S branch takes a single image as input and outputs its predicted segmentation map. The architecture of each network and the corresponding loss functions are detailed in Sections 2.3.

#### 2.1.1 G-R registration branch

The objective of the G-R branch is to predict a displacement field φ that maps a moving image *M* onto a fixed image *F*, both defined on the 3D spatial domain $\Omega \subseteq R^{3}$. Initially, the images *M* and *F* undergo separate feature extraction process via network G to produce their respective latent feature representation *Z_m_* and *Z_f_*. The training scheme detailed in Sections 2.2 ensures the modality-independence of extracted features, while also ensuring their commonality across both tasks. Subsequently, *Z_m_* and *Z_f_* are concatenated and fed to the registration network R. Network R learns the displacement field φ = *R*(*Z_f_*, *Z_m_*) between the two given common latent representations. The resulting φ is a 3D volume, where each voxel represents the offset from the corresponding point in the moving image to its counterpart in the fixed image along the *x*, *y*, and *z* directions. Finally, bilinear interpolation is employed to obtain the warped common representations *W(Z_m_*,φ*)*, and the same deformation is applied to the segmentation label *S_m_* with nearest neighbor interpolation to generate the warped label *W(S_m_*,φ*)*. Let *p* denote the coordinate of a given voxel in a moving image, for each voxel *p*∈Ω, the warping is defined as

(1) $$W\left(Z_{m},\varphi \right)[p]=Z_{m}[p+\varphi [p]],$$

It is worth noting that the registration process is performed in the latent feature space, and it is only during the test phase that we apply the learned displacement field to warp the moving images to obtain the warped images.

#### 2.1.2 G-S Segmentation branch

The G-S branch shares the feature extraction network G with the G-R branch. Since the segmentation network S takes a single image as input, the common latent features *Z_m_* and *Z_f_* generated by network G are individually passed to the network S to predict their segmentation masks ${\hat{S}}_{f}$ = S(*Z_f_*) and ${\hat{S}}_{m}$ = S(*Z_m_*). Similar to the registration network R, the segmentation network S also operates within the common latent feature space.

### 2.2 ESDR-guided two-stage training scheme

The core of the DCRS framework lies in the effective learning and utilization of common latent features. We expect the learned features to possess three characteristics: (i) task commonalities: the features should encapsulate the inherent correlations and commonalities between the registration and segmentation tasks in a simple and compact manner. (ii) Modality independence: to address the difficulties associated with multi-modal similarity metric design, we need the learned features to be invariant to imaging modality. (iii) Interpretability: rather than black-box learning, we aim for the learned features to have discernible physical meanings, thus facilitating the interpretation and manipulation for improved registration and segmentation.

Since image segmentation is essentially a classification problem, it takes a single image as input and outputs voxel-wise classification labels or confidence scores. On the other hand, image registration addresses a matching problem, wherein two images are taken as inputs to generate a displacement field that maps voxels in the moving image to corresponding voxels in the fixed image. The significant disparities in input and output characteristics between the two tasks, along with limited training data, make it difficult for direct data-driven learning of latent features to converge, and also cannot guarantee that the learned features meet the aforementioned requirements.

An intuitive observation about the commonality between registration and segmentation is that they share focus on brain region boundaries. While segmentation aims to precisely delineate the boundaries of interested brain regions, registration seeks to align these boundaries across images. However, simply offering boundary information falls short in delivering the intricate directional cues and distance specifics required for accurate displacement field generation, crucial in registration tasks. Similarly, it lacks the capability to accurately classify individual voxels as belonging to foreground or background regions, essential for achieving high-quality segmentation outcomes.

To meet our requirements on latent feature learning and effective network training, we propose an ESDR-guided two-stage training scheme. Firstly, based on our observation on the commonality between the two tasks, we introduce ESDR to explicitly encode the boundary and displacement information in images, and use it to pretrain the network G, S, and R separately. Subsequently, we concatenate the pretrained networks, and iteratively fine-tune the G-S and G-R branches to achieve task-level collaboration between the two tasks.

Given a 3D image stack and the segmentation label of its regions of interest (ROI) regions, the value of each voxel in the signed distance representation (SDR) is defined as the Euclidean distance from that voxel to the nearest boundary of the ROI in which it resides. Let *x* denote the spatial location of the given voxel, its corresponding value in the SDR is calculated as

(2) $$\text{SDR}(x)=\{\begin{array}{cc} \underset{y\in \tau }{\inf}||x-y|{|}_{2} & \;\text{if}\;x\in \Omega^{\mathrm{ROI}}\\ 0 & \;\text{if}\;x\in \tau \end{array},$$

where $\Omega^{\mathrm{ROI}}$ represents the ROI in the 3D brain image $I\subseteq R^{3}$. It is worth noting that background is treated as a separate ROI region. *τ* denotes the boundary of the ROI, and $||x-y|{|}_{2}$ denotes the Euclidean distance between the two points.

We can observe that the boundaries of brain regions are implicitly embedded as the zero level-sets of SDR, and the encoded voxel-to-boundary distance information offers compact clues for the efficient inference of voxel matching and the generation of displacement fields. In addition, the modality-dependent features including brightness, texture, noise, and artifacts are essentially decoupled and eliminated. This allows the segmentation network S and registration network R to focus only on the interested structural information of images, which significantly simplifies the model training and essentially eliminates the need for designing multi-modal similarity metrics. Benefiting from the interpretability of latent features, we can further enhance the attention of models to the boundaries and accelerate model training by applying an exponential transformation on the SDR:

(3) ESDR(x)=exp(−γ·SDR(x)).

The scalar parameter *γ* is used to regulate the impact of the exponential transformation, and we empirically set *γ* as 1. By amplifying the gradients at boundaries, ESDR not only reinforces the model’s focus on edge regions but also helps the gradient descent algorithm to converge faster during training.

**Figure 2.**
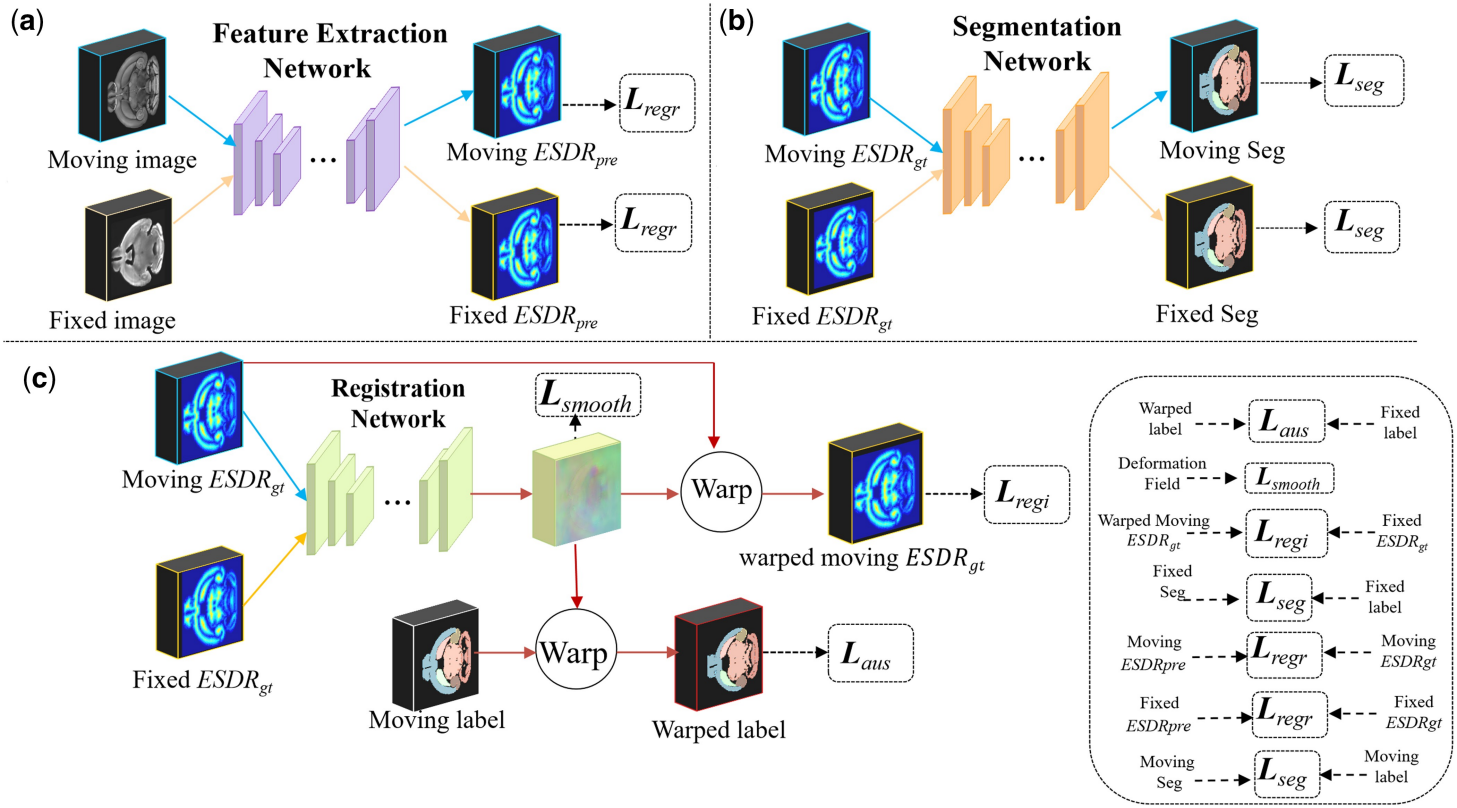
Flowchart of the three networks in the pre-training phase. (a), (b), and (c) denotes the pre-training process of feature extraction network, segmentation network, and registration network, respectively.

To guide and drive the effective latent feature learning, instead of training G-R and G-S branches from scratch, we use ESDR to pre-train the G, R, and S networks separately, followed by iterative fine-tuning of the G-R and G-S branches. Figure 2 illustrates the ESDR guided pre-training process of the three networks. During the pre-training phase, the feature extraction network G receives single image of different modalities as input and predicts their corresponding ESDR features. The ground-truth ESDR (*ESDR_gt_*) of an image can be generated directly from its segmentation labels using Equations 2 and 3. We minimize the discrepancy between the predicted ESDR (*ESDR_pre_*) and *ESDR_gt_* using the regression loss *L_regr_*, as detailed in Equation 4. The segmentation network S takes the *ESDR_gt_* of an image as input and is trained to minimize the difference between its predicted segmentation label and ground-truth label, as measured by *L_seg_*, shown in Equation (10). Meanwhile, the registration network R accepts the concatenated *ESDR_gt_* of paired images as input and produces a displacement field to minimize the difference between warped ESDR and fixed *ESDR_gt_* using *L_regi_*, as well as the difference between the warped label and the fixed label using *L_aus_*. The deformation field is regularized with the smooth loss *L_smooth_*. The loss functions *L_regi_*, *L_smooth_*, and *L_aus_* are detailed in Equations 5, 6, and 7, respectively.

In the fine-tuning phase, we concatenate the pre-trained networks G, S, and R to form G-S and G-R branches, and jointly optimize two branches in an iterative manner to achieve task-level collaboration of registration and segmentation. Unlike the pre-training phase, the inputs to both networks S and R are *ESDR_pre_* output by network G, rather than their ground-truth counterparts.

### 2.3 Structure and loss of networks

All networks G, S, and R follow the basic encoder-decoder architecture with skip connections similar to 3D U-Net (Çiçek *et al.* 2016). We use max-pooling for downsampling and deconvolution for upsampling. We detail the structure of the networks and their corresponding loss functions used in different training stages in this section.

#### 2.3.1 Feature extraction network

The feature extraction network G has five resolution stages, with each stage composed of two 3 × 3 × 3 convolution layers. The number of channels in the convolutional layers progressively increases across the stages, with 16, 32, 64, 128, and 256 channels in each respective stage. Each layer is followed by a group normalization (GN) and a rectified linear unit (ReLU).

In the pre-training phase, network G undergoes individual training to regress ESDR from input images, aiming to minimize the disparity *Diff* between the *ESDR_pre_* and the *ESDR_gt_*. We adopt Huber loss (Huber 1992) as the regression loss *L_regr_*:

(4) $$L_{\text{regr}}\left(\mathrm{Diff}\right)=\{\begin{array}{cc} \frac{1}{2}Diff^{2} & \left|\mathrm{Diff}\right|\leq \delta \\ \delta \cdot \mathrm{Diff}-\frac{1}{2}\delta^{2} & \left|\mathrm{Diff}\right|>\delta \end{array},$$

where $\mathrm{Diff}=\mathrm{ESD}R_{\mathrm{pre}}$−$\mathrm{ESD}R_{gt}$, and *δ* is a hyper parameter which determines how much to switch to MSE (Bauer and Kohavi 1999) loss(≤δ) or MAE loss (>δ). Smaller *δ* makes the loss function more robust to outliers, but may cause the model to converge more slowly in general. In our subsequent experiments, *δ* takes the value of 1.

#### 2.3.2 Registration network

The registration network R has four resolution stages, with each stage composed of one 3 × 3 × 3 convolution layer. There are 16, 32, 32, 32 channels in the convolutional layer of each stage. Except for the last convolution layer, each other convolution layer is followed by a ReLU layer. The total loss function of Network R is composed of the registration loss *L_regi_*, smooth loss *L_smooth_* and auxiliary loss *L_aus_*.

The registration loss *L_regi_* is used to measure the voxel-wise mean squared error between the ESDR of fixed image (*ESDR_f_*) and the warped ESDR of moving image (*ESDR_m_*). It is worth noting that since ESDR is inherently modality independent, we can adopt a simple MSE metric (Bauer and Kohavi 1999) as the registration loss for both multi-modal and mono-modal tasks.

(5) $$L_{\text{regi}}=\frac{1}{\Omega }\sum_{p\in \Omega }{\left[\mathrm{ESD}R_{f}(p)-W\left(\mathrm{ESD}R_{m},\varphi \right)(p)\right]}^{2}.$$

The loss function *L_smooth_* is calculated to constrain the smoothness of the deformation field φ. Following the previous method (Balakrishnan *et al.* 2019), we use L2 regularization as the deformation smooth loss, and *p* represents the voxels in the image. The loss is defined as

(6) $$L_{\text{smooth}}=\sum_{p\in \Omega }||\nabla \varphi (p)|{|}_{2}^{2},$$

where $\nabla \varphi (p)=\left(\frac{\partial \varphi (p)}{\partial (x,y,z)}\right)$, we approximate $\frac{\partial \varphi (p)}{\partial (x,y,z)}\approx \varphi \left(p_{\left\{x,y,z\right\}}+1\right)-\varphi \left(p_{\left\{x,y,z\right\}}\right)$.

The auxiliary loss *L_aus_* calculates the overlap between the warped moving label $S_{m}^{w}=S_{m}{}^{\circ}\varphi$ and the fixed label *S_f_* to further assist the registration network to improve the global correspondence of anatomical structures. Auxiliary loss *L_aus_* for all structures k ∈ [1, K] is defined as:

(7) $$L_{\text{aus}}\left(S_{f}^{k},S_{m}^{k}{}^{\circ}\varphi \right)=1-\frac{1}{k}\sum_{k=1}^{k}\text{Dice}\left(S_{f}^{k},S_{m}^{k}{}^{\circ}\varphi \right).$$

Let $S_{f}^{k},\;S_{m}^{k}{}^{\circ}\varphi$ represent the k-th structure for *S_f_* and $S_{m}^{w}$, respectively. Dice (1945) loss is used to evaluate the volume overlap for structure *k*, which is formulated as

(8) $$\text{Dice}\left(S_{f}^{k},S_{m}^{k}{}^{\circ}\varphi \right)=2\cdot \frac{\left|S_{f}^{k}\cap S_{m}^{k}{}^{\circ}\varphi \right|}{\left|S_{f}^{k}\right|+\left|S_{m}^{k}{}^{\circ}\varphi \right|}.$$

With the above three defined loss functions, the total loss function to train the network R is defined as

(9) $$L_{R}=\alpha L_{\text{regi}}+\beta L_{\text{smooth}}+\rho L_{\text{aus}\;}.$$

It is worth noting that during the pretraining phase, *ESDR_f_* and *ESDR_m_* are calculated from the given segmented labeling. While in the fine-tuning phase, as network R is concatenated behind network G, the *ESDR_f_* and *ESDR_m_* correspond to the outputs generated by network G. The entire G-R branch is fine-tuned by minimizing *L_R_*.

#### 2.3.3 Segmentation network

The segmentation network S is composed of four resolution stages. Each stage comprises two 3 × 3 × 3 convolution layers. The first convolutional layer of each stage has 8, 16, 32, and 64 channels. The second convolutional layer in each stage has twice the number of convolutional channels as its preceding layer. Each layer is followed by a GN and a Leaky ReLU. In the final layer, a 1 × 1 × 1 convolution is applied to estimate the one-hot representation of segmentation labels with N channels.

We define the segmentation loss *L_seg_* to maximize the overlap between predicted segmentation masks and their corresponding ground-truth labels:

(10) $$L_{\mathrm{seg}}\left({\hat{S}}^{k}(p),S^{k}(p)\right)=1-\frac{1}{k}\sum_{k=1}^{k}\frac{2\cdot \left|{\hat{S}}^{k}(p)\cap S^{k}(p)\right|}{\left|{\hat{S}}^{k}(p)\right|+\left|S^{k}(p)\right|},$$

where ${\hat{S}}^{k}$ and *S^k^* represent the one-hot encoding of *k*th class structure for predicted segmentation mask $\hat{S}$ and ground-truth label *S*.

In the pretraining phase, we use *L_seg_* to drive the training of the segmentation network. In the fine-tuning phase, the same *L_seg_* is used to train the entire G-S branch.

## 3 Experiment configurations

### 3.1 Dataset

We evaluate the multi-modal registration and segmentation performance of our DCRS framework on whole-brain dataset of mouse that contain three imaging modalities: STPS, fMOST, and VISoR. Furthermore, to demonstrate the generalization of our method, we extend our analysis to encompass two mono-modal human brain datasets acquired through Magnetic Resonance Imaging (MRI). We assume affine alignment of the fixed and moving images at the preprocessing stage. To enhance the diversity and quantity of the dataset, we employed the deep structure sampling method (He *et al.* 2020) to augment the data for all of the following datasets.

#### 3.1.1 Multi-modal mouse brain datasets

This dataset comprises the average and annotation template of Allen CCFv3, available for download from the Allen Institute web portal (http://atlas.brain-map.org/), along with mouse brain images from three modalities: STPT (Economo *et al.* 2016), VISoR (Xu *et al.* 2021), and fMOST (Gong *et al.* 2016). There are 33, 60, and 43 labeled images in the STPT, VISoR, and fMOST subsets, respectively. Eight major mouse brain areas were selected as ROIs. All images in the dataset are globally aligned to the same anatomical template using the mBrainAligner (Qu *et al.* 2022) and resampled to 134 × 100 × 162. For the three multi-modal registration tasks (CCF-fMOST, CCF-VISoR, and fMOST-VISoR), we generated 240 pairs of data for each task, respectively. We randomly selected 200 pairs of images as the training set and kept the remaining 40 pairs as the test set.

#### 3.1.2 Mono-modal human brain datasets

**LPBA40 (** Shattuck *et al.* 2008 **):**

The LPBA40 dataset comprises 40 labeled MRI brains, with annotations delineating fifty-four clinical ROIs. All these regions were chosen as the ROIs in our experiments. To accommodate the network’s input size requirements, we resampled these images to dimensions of 160 × 192 × 160. Through data augmentation, we expanded the dataset to include a total of 270 brain images, with 216 images allocated for the training set and 54 images for the test set.

**Mindboggle (** Klein and Tourville 2012 **):**

Mindboggle constitutes an MRI brain dataset with labeled 35 cortical structures, encompassing a total of 82 3D images from various datasets, including OASIS-TRT-20, MMRR-21, and HLN-12. We merge 35 cortical labelings into 8 labelings as the brain ROIs. These images were resampled to 160 × 192 × 160 to fit the network. We augment the dataset to 264 images with 216 images selected for the training set and 48 images for the test set.

### 3.2 Implementation details

We implement our DCRS in PyTorch on a single NVIDIA Tesla GPU with 24G memory. All DL-based models are optimized using Adam optimizer (Kingma and Ba 2014) with an initial learning rate of $10^{-3}$. The training batch size is set to 1 to save memory, and we train all models for 160 epochs. The weight values of all loss functions were set to 1.

### 3.3 Comparison settings

To demonstrate the superiority of our method, we compare DCRS against eight widely-used registration methods. This comparison includes two traditional methods: BSpline (Rueckert *et al.* 1999) and SyN (Avants *et al.* 2008), as well as six DL-based methods: LC-VoxelMorph-Mind (VM-Mind), LC-VoxelMorph-SSIM (VM-SSIM), LC-VoxelMorph-LNCC (VM-LNCC), Deepatlas (Xu and Niethammer 2019), PC-Reg-RT (He *et al.* 2021), and LC-TransMorph (Chen *et al.* 2022). The prefix “LC-” denotes the “label-constrainted” variants of VoxelMorph and TransMorph, while the suffixes “-Mind,” “-SSIM,” and “-LNCC” denote the use of MIND (Heinrich *et al.* 2012), SSIM (Wang *et al.* 2004), and LNCC (Balakrishnan *et al.* 2019) as similarity metrics, respectively. For simplicity, we abbreviate LC-VoxelMorph as VM. LC-TransMorph employs SSIM and Dice loss for network training. VM-LNCC, DeepAtlas, and PC-Reg-RT are served as mono-modal registration baselines for evaluating the generalization of DCRS. DeepAtlas and PC-Reg-RT were evaluated with default parameters. Additionally, we compared our results with a standard 3D U-Net (Çiçek *et al.* 2016) to highlight improvements in segmentation performance. To ensure fairness, all DL-based methods were trained and tested on the same datasets, while non-DL methods were evaluated on the same test set without training. Detailed configurations for BSpline and SyN are provided in the Supplementary Material.

### 3.4 Evaluation metric

We use Dice similarity coefficient (DSC)(%) between the warped label and fixed label to assess the registration performance. Higher DSC score indicates better accuracy of registration. For image registration task, deformations field should be realistic without the folding. Therefore, we quantify the smooth of displacement field φ using the Jacobian matrix $J\varphi (p)=\nabla \varphi (p)\in R^{3\times 3}$. We count the negative voxels defined by ∇φ(p)≤0 in each volume. Then, we calculated the fraction of Jφ(p)≤0 [%] for each DL-based method to quantitatively measure the smoothness of the displacement field. Generally speaking, a lower number of negative Jacobian determinants indicates better smoothness of the displacement field. The standard deviation (std) of these metrics is also provided to evaluate the stability of models. We compute the DSC [%] between the predicted and ground-truth segmentation masks to measure the accuracy of segmentation.

### 3.5 Result and discussion

We systematically assessed the proposed methods through both quantitative and qualitative analyses using multi-modal mouse brain dataset and mono-modal human brain dataset.

#### 3.5.1 Quantitative evaluation

We evaluated the effectiveness of the proposed DCRS on three multi-modal registration datasets CCF-fMOST, CCF-VISoR, and VISoR-fMOST, which contain the eight labeled major brain regions selected as ROIs, including hypothalamus (HY), caudoputamen (CP-1/CP-2), hippocampal formation (HPF-1/HPF-2), cerebral cortex (CTX), cerebellar cortex (CBX), and brain stem (BS). For all datasets, we calculate the DSC on all datasets to evaluate the registration and segmentation accuracy of different methods. The registration and segmentation results are shown in Table 1.

**Table 1.** Quantitative comparison in terms of Reg-DSC, Seg-DSC, and ∇|J|≤0 (%) on CCF-fMOST, CCF-VISoR, and VISoR-fMOST mouse brain dataset, where “Reg-DSC” and “Seg-DSC” represent the DSC for registration and segmentation tasks, black bold represents the optimal result of all comparison algorithms for every metric.

Method	Reg-DSC(%)	∇|J|≤0 **(%)**	Seg-DSC(%)
(a) Mouse Brain CCF-fMOST multi-model registration			
Global only	70.17		
BSpline	73.17 ± 12.79	0	
SyN	87.81 ± 2.26	0	
VM_Mind	84.40 ± 2.00	0.107 ± 0.017	
VM_SSIM	84.16 ± 2.92	**0.07 ± 0.026**	
VM_LNCC	84.04 ± 2.44	0.152 ± 0.046	–
LC-TransMorph	72.15 ± 2.15	0.204 ± 0.141	–
DCRS (Ours)	**90.48 ± 0.512**	0.137 ± 0.022	92.6
3D U-Net			80.05
(b) Mouse brain CCF-VISoR multi-model registration			
Global only	82.04		
BSpline	83.83 ± 6.39	0	
SyN	87.43 ± 3.78	0	
VM_Mind	85.65 ± 1.41	0.083 ± 0.039	
VM_SSIM	87.56 ± 1.59	1.03 ± 0.344	
VM_LNCC	85.65 ± 1.33	0.15 ± 0.10	
LC-TransMorph	84.78 ± 0.588	0.034 ± 0.023	
DCRS (Ours)	**92.49 ± 0.522**	**0.031 ± 0**	93.67
3D U-Net			89.34
(c) Mouse Brain VISoR-fMOST multi-model registration			
Global only	70.8		
BSpline	73.66 ± 6.78	0	
SyN	71.77 ± 6.78	0	
VM_Mind	82.18 ± 2.16	**0.051 ± 0.029**	
VM_SSIM	82.67 ± 1.60	0.09 ± 0.05	
VM_LNCC	77.07 ± 1.67	0.96 ± 0.273	
LC-TransMorph	74.12 ± 3.43	0.273 ± 0.156	
DCRS (Ours)	**86.69 ± 0.85**	0.176 ± 0.043	93.24
3D U-Net			85.04

As shown in Table 1, our DCRS achieves Reg-DSC of 90.48% (a), 92.49% (b), 86.69% (c), and Seg-DSC of 92.6% (a), 93.67% (b) and 93.24% (c) for CCF-fMOST, CCF-ViSoR, and ViSoR-fMOST datasets. Compared with VM_Mind, our DCRS exhibits notable improvements in Reg-DSC by 6.08% (a), 6.84% (b), and 4.51% (c), respectively. Compared to LC-TransMorph, our DCRS improves by 18.33% (a), 7.71% (b), and 12.57% (c) for CCF-fMOST, CCF-ViSoR, and ViSoR-fMOST datasets on Reg-DSC. These results illustrate the effectiveness of our modality-independent latent representation learning for both multi-modal registration and segmentation tasks.

We also assess the generalization of our method on two mono-modal human brain datasets LPBA40 and Mindboggle, and results are shown in Supplementary Table S2. Our DCRS achieves the highest Reg-DSC among the seven compared algorithms. Further detailed analyses are available in the Supplementary Material.

**Figure 3.**
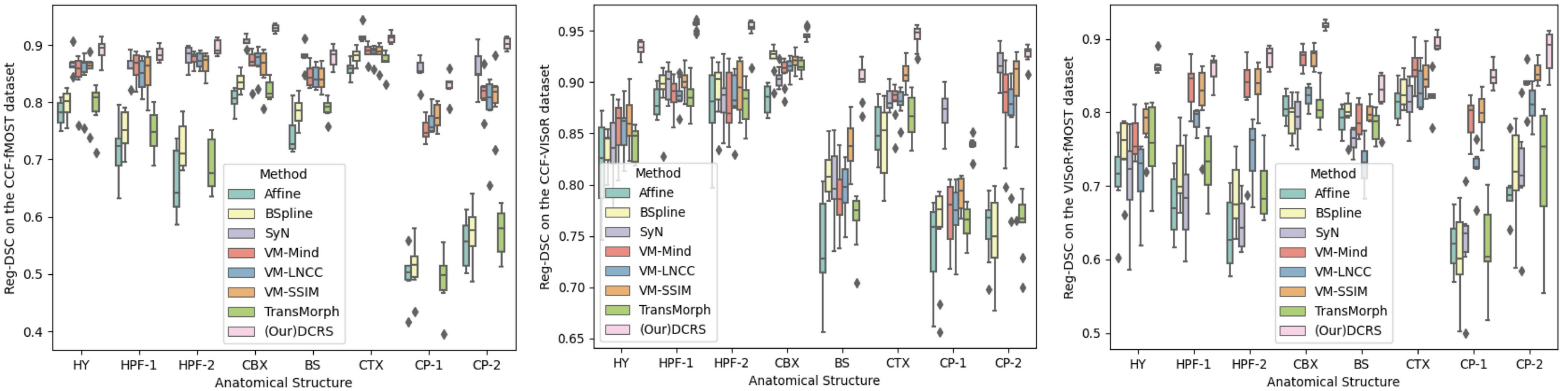
The comparison of multi-modal registration performance of different methods on eight brain regions of mouse brain.

In Figure 3, we show the boxplot of Reg-DSC scores of various registration methods across different brain structures on the CCF-fMOST, CCF-VISoR, and fMOST-VISoR datasets. The last pink box in each brain region highlights the competitive registration performance achieved by our method across all structures. It can be observed that, compared with competing methods, our method not only demonstrates superior registration accuracy but also exhibits enhanced stability across diverse brain regions. The shorter box lines and fewer outliers associated with our method underscore its robust performance and stability across various anatomical structures.

#### 3.5.2 Qualitative evaluation

**Figure 4.**
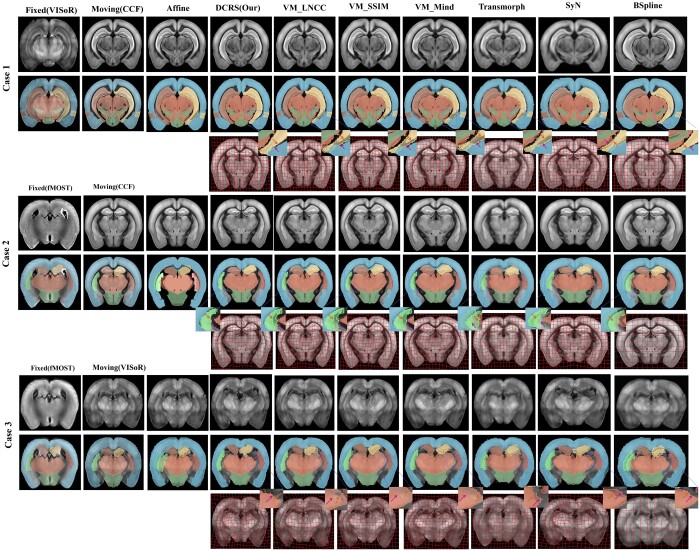
Visual comparison of registration accuracy of different methods on the CCF-VISoR, CCF-fMOST, and VISoR-fMOST datasets. For each case, the first row shows the raw input and registered images. The second row visualizes the segmentation labels with bright colors to facilitate comparison of registration accuracy of different brain regions. The third row depicts the displacement field generated by different methods. The arrows in the magnifying views point to the boundary lines of the fixed label and warped label, respectively. Ideally, they should overlap.

We visualize the registration results of different methods and their corresponding displacement field in Fig. 4. Cases 1–3 show comparative results on the CCF-VISoR, CCF-fMOST, and fMOST-VISoR tasks, respectively, along with results overlaid with warped segmentation labels and displacement field. We also display segmentation results on the fMOST dataset with three different methods: 3D U-Net, our DCRS, and ground truth in Supplementary Fig. S1.

In case 1 (Fig. 4), it is evident that several DL-based methods (VM-Mind, VM-SSIM, and VM-LNCC) are unable to precisely align the hippocampal (HPF-1/HPF-2) regions (highlighted in yellow and light red). This difficulty arises from the multimodal nature of the images, which hinders these DL-based methods from effectively extracting voxel-corresponding features. In contrast, our DCRS achieves superior performance by leveraging the strong prior guidance provided by ESDR, leading to improved perception and alignment of edges. While VM-Mind, VM-SSIM, and VM-LNCC demonstrate synergy between segmentation and registration at the task level, our DCRS method goes a step further by coupling at both the task and feature levels. This distinctive approach enhances the stability of our algorithms in aligning diverse brain regions, thereby minimizing distortions effectively. In case 2, our method exhibits better accuracy in the CP-1 (green), and in case 3, our DCRS is more accurate than other method in CTX (brick red). The visual comparison results are consistent with quantitative results, further validating the effectiveness of our DCRS.

## 4 Ablation experiment

To evaluate the impact of each component of DCRS on the performance of registration and segmentation, we have conducted the ablation studies on the VISoR-fMOST dataset, and the results are presented in Table 2. First, we assess the performance of DCRS with and without ESDR guided two-stage training. It can be observed that the introduction of ESDR guided training led to a substantial improvement in Reg-DSC by 11.75%, and noticeable enhancements in segmentation performance can also be observed. We further investigate the performance of DCRS without applying the exponential transformation (ET) on the SDR. Notably, the exponential transformation contributed to 3.35% improvement in Reg-DSC. In addition, the introduction of exponential transformation also significantly accelerate the training speed, we provided the loss convergence plot of our DCRS with and without exponential transformation in the Supplementary Fig. S2.

**Table 2.** Ablation experiment results, where “w/o” represents “without,” “f” represents “fMOST,” and “V” represents “VISoR”

Method	Reg-DSC (%)	Seg-DSC (%)(f)	Seg-DSC (%)(V)
DCRS (w/o ESDR)	74.94	90.8	93.1
DCRS (w/o ET)	83.34	91.13	93.22
DCRS (ours)	86.69	92.69	93.79

## 5 Modality-independence and interpretability of latent features

**Figure 5.**
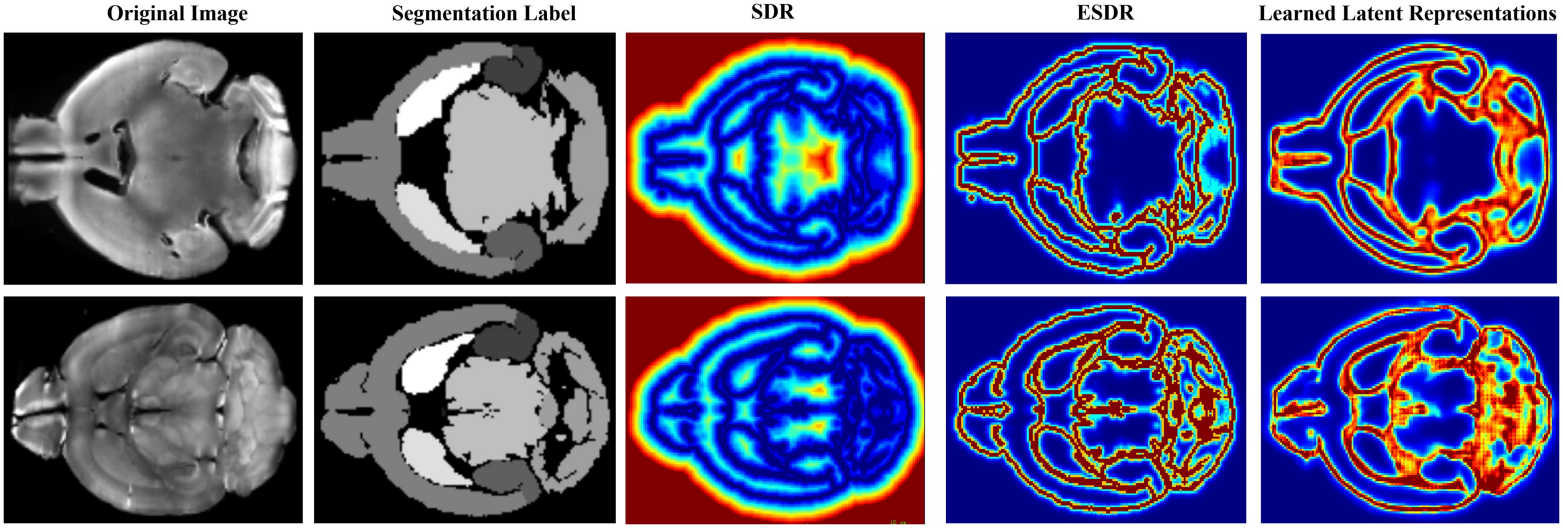
Illustration of modality-independence and interpretability of learned latent features. The first and second rows show the original images, segmentation labels, SDR, ESDR, and learned representations for the mouse brain images captured using the fMOST and VISoR modalities, respectively.

In the pre-training phase, we use ESDR to guide and promote the modality independence and interpretability of learned latent features. To verify the preservation of these characteristics after fine-tuning, we visualize in Fig. 5, the latent features learned from two images of different modalities using the fine-tuned feature extraction network. Firstly, we observe that learned latent features effectively capture the anatomical boundary information of ROIs across modalities. Notably, modality-dependent features such as bright distributions and textures are effectively decoupled and eliminated, confirming the modality-independent feature extraction capability of our network. Secondly, the learned latent features closely resemble the appearance of ESDR. Given that ESDR is designed with explicit physical meanings, specifically the exponential signed distance representation of images, we can reasonably infer the interpretability of the learned latent features. Finally, the resemblance of the learned latent features to ESDR rather than SDR indicates our ability to intuitively manipulate feature learning within the DCRS framework.

## 6 Discussion

In this study, we introduced the DCRS framework to address the challenges inherent in multimodal whole-brain image registration and segmentation. By deeply coupling the two tasks and leveraging modality independent latent feature representations, DCRS effectively bypasses the complexities of designing cross-modal similarity metrics and enhances the performance of both tasks simultaneously. Additionally, the introduction of ESDR also ensures interpretability of learned features, promoting effective latent feature learning. While DCRS demonstrates superior performance in mouse and human brain datasets for both registration and segmentation tasks, it is important to acknowledge its limitation: DCRS predominantly operates at the structural level, assuming smooth deformation fields within labeled anatomical structures. Although this assumption is generally applicable in biological imaging, it may limit the method’s ability to capture finer-scale internal deformations or non-uniform variations within specific brain regions. To address this limitation, one simple solution is to provide more detailed labels for finer anatomical regions of interest. This would enable DCRS to capture and align intricate anatomical variations with higher precision. One promising direction for future research involves integrating structure-level registration within DCRS with pixel-level registration techniques. Such a hybrid approach is expected to provide enhanced granularity, allowing for the precise alignment of finer details that are currently overlooked by structural-level methods.

## Supplementary Material

btae606_Supplementary_Data
