# Supplementary material for: Deep coupled registration and segmentation of multimodal whole-brain images
Source: Bioinformatics. 2024 Oct 14;40(11):btae606. doi: 10.1093/bioinformatics/btae606 (PMC11543610; doi:10.1093/bioinformatics/btae606)
Supplement: btae606_Supplementary_Data [file btae606_supplementary_data.pdf]

Comparison Settings

BSpline is implemented by Elastix (Klein, et al., 2009; Shamonin, et al., 2014) and tuned. For the three multi-modal registration tasks, the main parameters of the BSpline fine-tuning are detailed in Supplementary Table 1. SyN was implemented using the publicly available Advanced Normalization Tools (ANTs)(Avants, et al., 2011) with default parameters, we chose mutual information as similarity metrics.

Supplementary Table 1 Main parameters of BSpline fine tuning for three multi-modal registration tasks

CCF-FMOST	CCF-ViSoR	fMOST-ViSoR
(FixedInternalImagePixelType "float")<br>(MovingInternalImagePixelType "float")<br>(UseDirectionCosines "true")<br>//**** Transformation **<br>(FinalGridSpacingInPhysicalUnits 16)<br>(HowToCombineTransforms "Compose")<br>//***** Similarity measure **<br>(NumberOfHistogramBins 32)<br>(ErodeMask "false")<br>// **** Multiresolution ****<br>(NumberOfResolutions 4)<br>// *** Optimizer *******<br>(MaximumNumberOfIterations 500)<br>// **** Image sampling **<br>(NumberOfSpatialSamples 2048)<br>(NewSamplesEveryIteration "true")<br>(ImageSampler "Random")<br>// **** Interpolation and Resampling ***<br>(BSplineInterpolationOrder 1)<br>(FinalBSplineInterpolationOrder 3)<br>(DefaultPixelValue 0)<br>(WriteResultImage "true")<br>(ResultImagePixelType "short")<br>(ResultImageFormat "mhd")	(FixedInternalImagePixelType "float")<br>(MovingInternalImagePixelType "float")<br>(FixedImageDimension 3)<br>(MovingImageDimension 3)<br>(UseDirectionCosines "true")<br>// **** Transformation *****<br>(FinalGridSpacingInVoxels 8 8 8)<br>(HowToCombineTransforms "Compose")<br>// **** Similarity measure ***<br>(NumberOfHistogramBins 32)<br>(ErodeMask "false")<br>(Metric0Weight 1.0)<br>(Metric1Weight 5.0)<br>// *** Multiresolution **<br>(NumberOfResolutions 2)<br>(ImagePyramidSchedule 1 1 1 1 1 1 1 1 1 1 1 1)<br>//*** Optimizer ***<br>(MaximumNumberOfIterations 1000)<br>(MaximumStepLength 0.0365)<br>// *** Image sampling **<br>(NumberOfSpatialSamples 2048)<br>(NewSamplesEveryIteration "true")<br>(ImageSampler "Random")<br>//*** Interpolation and Resampling *<br>(BSplineInterpolationOrder 1)<br>(FinalBSplineInterpolationOrder 3)<br>(DefaultPixelValue 0)	(FixedInternalImagePixelType "float")<br>(MovingInternalImagePixelType "float")<br>(FixedImageDimension 3)<br>(MovingImageDimension 3)<br>(UseDirectionCosines "true")<br>// ***** Transformation ****<br>(FinalGridSpacingInVoxels 8 8 8)<br>(HowToCombineTransforms "Compose")<br>// **** Similarity measure ***<br>(NumberOfHistogramBins 32)<br>(ErodeMask "false")<br>(Metric0Weight 1.0)<br>(Metric1Weight 0.3)<br>//***** Multiresolution ***<br>(NumberOfResolutions 2)<br>(ImagePyramidSchedule 1 1 1 1 1 1 1 1 1 1 1 1)<br>//******* Optimizer *********<br>(MaximumNumberOfIterations 1000)<br>(MaximumStepLength 0.0365)<br>// ** Image sampling ***<br>(NumberOfSpatialSamples 2048)<br>(NewSamplesEveryIteration "true")<br>(ImageSampler "Random")<br>// **** Interpolation and Resampling **<br>(BSplineInterpolationOrder 1)<br>(FinalBSplineInterpolationOrder 3)<br>(DefaultPixelValue 0)

	(WriteResultImage "true")<br>(ResultImagePixelType "float")<br>(ResultImageFormat "mhd")	(WriteResultImage "true")<br>(ResultImagePixelType "float")<br>(ResultImageFormat "mhd")

As shown in Supplementary Table 2, on the LPBA40 and Mindboggle datasets, our Reg-DSC reaches 84.3% and 83.79%. Compared to other methods that employ joint segmentation and registration strategies, such as DeepAtlas, and PC-reg-RT, the Reg-DSC of DCRS improves by 13.96%(d), 11%(d), and 8.66%(e), 5.09%(e). In addition to the superior registration accuracy, our DCRS also has a stable ∇||Jφ||≤ 0(%). Our DCRS achieve ||Jφ||≤ 0(%) of 0.6%(d), 0.217%(e), which is 1.627%(d) and 5.45%(e) smaller than VM_LNCC (λ=0.1), respectively, indicating that our algorithms can obtain a reasonable and smooth displacement field. In addition, the deformation smoothness obtained by PC-Reg-RT on the Mindboggle dataset is slightly higher than that of our method. However, the registration accuracy of our method is much higher than that of PC-Reg-RT.

Supplementary Table 2 Quantitative comparison of registration and segmentation in terms of DSC, ∇|J|≤ 0(%) on LPBA40 and Mindboggle.

Method	Reg-DSC(%)	∇|J|≤ 0(%)	Seg-DSC(%)
d) Human Brain(LPBA40) MRI mono-model registration			
Affine only	63.17	-	-
SyN	68.98±7.3	0	-
Bspline	69.24±7.2	0	-
VM_LNCC(λ=1)	75.64±0.947	0.437±0.045	-
VM_LNCC(λ=0.1)	76.22±0.46	2.227±0.165	-
DeepAtlas	70.34±0.035	**0.244±0.025**	85.61±0.02
PC-Reg-RT	73.3±0.067	0.97±0.31	87.671±0.03
DCRS (Ours)	**84.3±0.97**	0.60±0.053	85.91±0.003
e) Human Brain(Mindboggle) MRI mono-model registration			
Affine only	62.6	-	-
SyN	74.64±0.073	0	-
Bspline	75.15±0.063	0	-
VM_LNCC(λ=1)	77.66±2.177	1.259±0.166	-
VM_LNCC(λ=0.1)	79.01±2.120	5.677±0.355	-
DeepAtlas	75.13±0.056	1.67±0.132	87.32±0.025
PC-Reg-RT	78.7±0.089	**0.022±0.001**	88.774±0.05
DCRS (Ours)	**83.79±2.02**	0.217±0.029	87.92±0.002

As shown in Supplementary Figure 1, we can observe that DCRS can produce results much more similar to the Ground-Truth than 3D UNet in terms of the HY, CBX, CP1, and CP2 regions by red boxes. Specifically, our DCRS cares more about the anatomical boundaries and outputs more accurate segmentation due to the feature extraction network partially pre-extracts anatomical priori information about each brain region, which is crucial for the segmentation task, while 3D UNet ignores these boundary details.

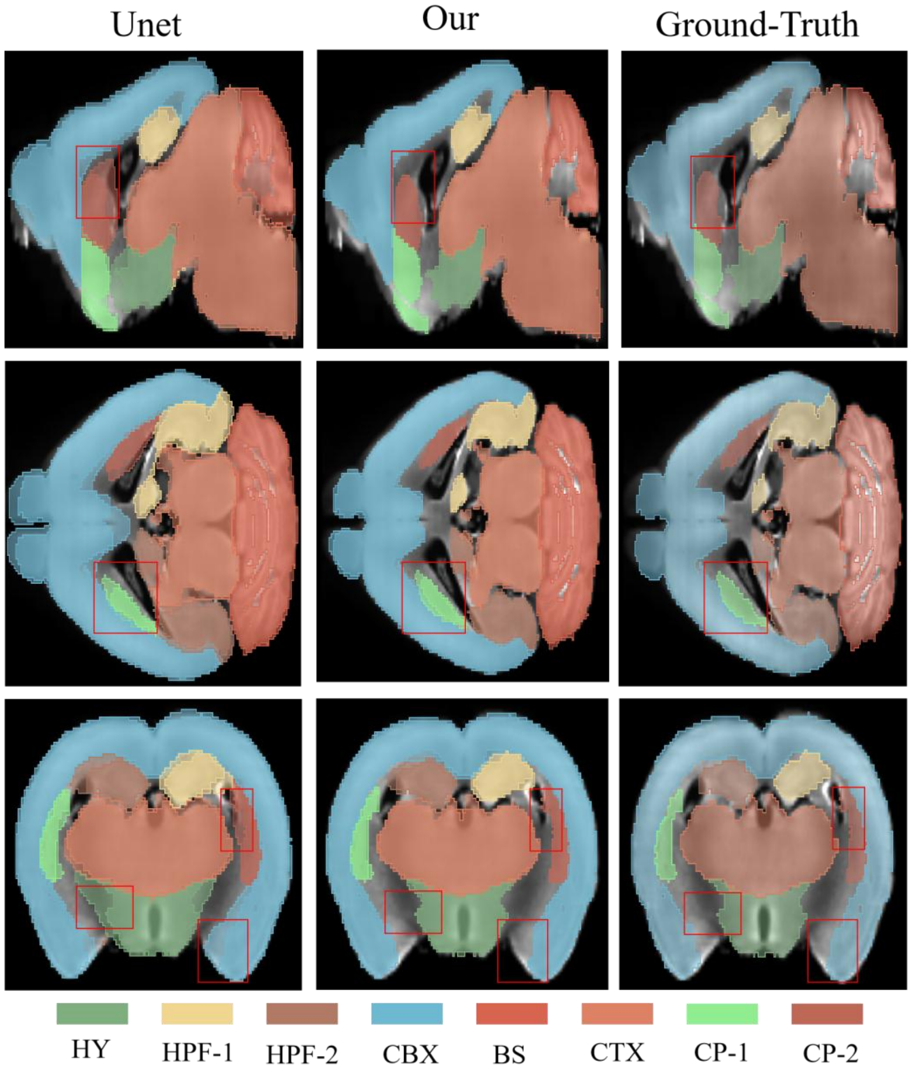

Supplementary Figure 1 Visual comparison of segmentation accuracy on fMOST dataset with two methods: 3D Unet, DCRS(Our). The top, middle, and bottom rows are the sagittal plane, cross-section, and coronal, respectively.

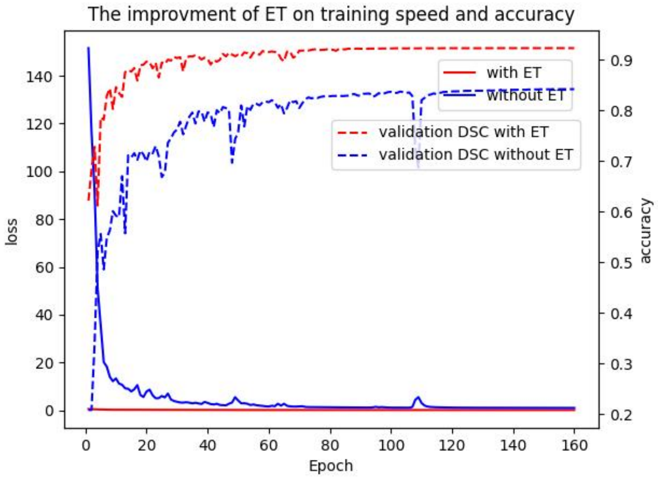

Supplementary Figure 2 Loss convergence curves and validation DSC curves with/without exponential transformation

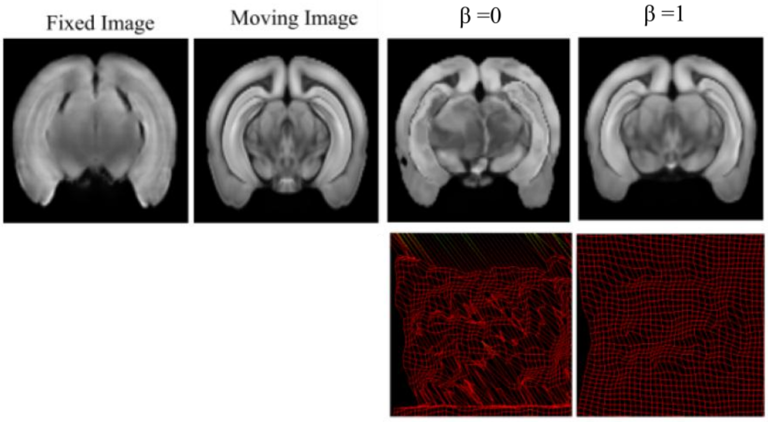

Supplementary Figure 3 The effect of L_{smooth} on the deformation field is added during training. The first column shows the fixed images, and the second column displays the moving images. The third and fourth columns present the visulization result without L_{smooth} ($\beta = 0$) and with L_{smooth} ($\beta = 1$), respectively， where first row contains the warped images, and the second row illustrates the deformation fields.

Reference

Avants, B.B., *et al.* A reproducible evaluation of ANTs similarity metric performance in brain image registration. *Neuroimage* 2011;54(3):2033-2044 %@ 1053-8119.

Klein, S., *et al.* Elastix: a toolbox for intensity-based medical image registration. *IEEE transactions on medical imaging* 2009;29(1):196-205 %@ 0278-0062.

Shamonin, D.P., *et al.* Fast parallel image registration on CPU and GPU for diagnostic classification of Alzheimer's disease. *Frontiers in neuroinformatics* 2014;7:50 %@ 1662-5196.
